# Forme ulcéro-nécrotique d’un cancer de la vulve

**DOI:** 10.11604/pamj.2017.27.130.11949

**Published:** 2017-06-19

**Authors:** Amel Achour Jenayah, Abdallah Cherni

**Affiliations:** 1 Service “A” du Centre de Maternité et de Néonatologie de Tunis, Tunisie

**Keywords:** Carcinome épidermoide, grande lèvre, forme ulcéro-nécrotique, Vulvar carcinoma, labia majora, ulceronecrotic cancer

## Image en médecine

Le cancer de la vulve est une affection néoplasique peu fréquente représentant moins de 5% des cancers gynécologiques, il survient le plus souvent chez des femmes âgées. Notre patiente est âgée de 70 ans. La symptomatologie clinique était dominée par un prurit vulvaire évoluant depuis 6 ans, négligé par la patiente. La lésion touche de manière préférentielle les grandes lèvres, c'est le cas de notre patiente qui présentait une forme ulcéro-nécrotique qui a touché la grande lèvre droite puis s'est étendu à la petite lèvre homolatérale. Le diagnostic était posé après la réalisation d'une biopsie vulvaire qui avait conclu à un carcinome épidermoïde, type histologique le plus fréquent. L'IRM abdomino-pelvienne avait classé la tumeur selon la classification FIGO 2009 en stade III A. La patiente avait bénéficié d'une vulvectomie élargie associée à une lymphadénectomie inguinale bilatérale suivie d'une radiothérapie externe.

**Figure 1:**
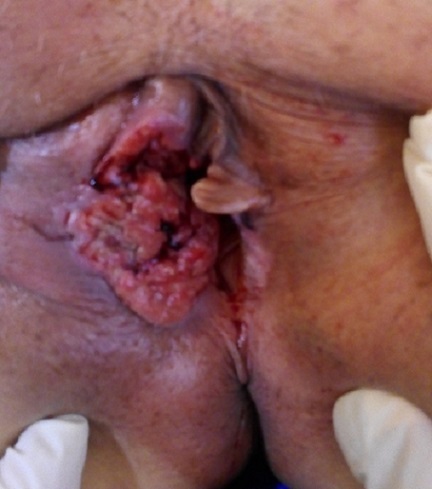
Aspect clinique de la lésion ulcéro-nécrotique de la grande lèvre droite étendue à la petite lèvre homolatérale

